# Reviews and Notices

**Published:** 1886-11-13

**Authors:** 


### Reviews and Notices.

*Practical Medicine*. By Sir James Sawyer, M.D., etc. Birmingham: Cornish Brothers, 1886.

This book well indicates the position attained by modern medicine, a position of scientific honesty and of practical endeavour. In no science or art has the teaching of Bacon been more fruitful than in that of the physician. Observations, facts, experiments—these are the weapons which have taken the place of crudities, mysteries, and occult influences in the armoury of him who in these days fights disease and death. The modern physician does not deal in mystery, and as little as he can in improved theory. He is watchful, experimental, tentative, hopeful, and has great faith in the "vis medicatrix naturæ," provided the "vis" has fair play. A great part of his business is to remove impediments, and give all possible encouragement to that same beneficent "vis." It is impossible in the space at our disposal to furnish anything like an adequate popular view of the varied contents of this sober and solid little book. Although the subjects discussed are comparatively few they cover a great deal of ground, and include some of the most important and difficult questions in practical medicine. Insomnia, intestinal obstruction, phthisis, cardiac disease, floating kidney—these and kindred topics require for their understanding and intelligent presentation an amount of labour, thought, and experience of no common degree. All these subjects are treated with skill and judgment. For the benefit of the student and the unprofessional reader the first of them will be considered a little more in detail, and from the quality of the argument on this subject the value of the general contents of the book may be estimated.

Insomnia, or sleeplessness, is the subject of the first lecture. Sleeplessness, unhappily, is by no means uncommon. There are few intellectually active persons of more than forty years of age who have not had more or less experience of its baneful companionship. Now this symptom, according to Sir J. Sawyer, is not an accident, but a something which drops from the clouds, which can neither be understood nor explained. It is a recognised effect of certain well-known causes, and is often quite under the control of the person who suffers from it. It is the merit of this lecture to have given a clear and comprehensible account of it; to have explained its immediate or physiological cause, its remote or efficient cause or causes, and the steps to be taken for its mitigation, its prevention, or its cure. In by far the larger proportion of instances the cause of sleeplessness is either work or worry. Less frequently, but by no means seldom, its symptom is brought on by the unwise use of alcohol or tobacco. Well, but over these causes the sleepless man must be admitted to have a good deal of control, at any rate in the use of tobacco or alcohol. The discontinuance or modified use of these is often sufficient to effect a complete cure. Even when overwork or worry produces sleeplessness, the patient frequently has the cure in his own hands. Some change in his habits, some diminution of his expenses, some curtailment of his labours is demanded, and this being well understood, the change is made, and the reward of sound and refreshing slumber is secured.

A lecture of this kind, though primarily intended for medical men, cannot but be of great interest to the large class who are or have been victims to sleeplessness. It is, as far as possible, free from technicalities, clear and simple in style, and, to those whose experience has given them some understanding of the subject, at least as interesting as a good many novels.

The whole volume is of the same practical and intelligent character, and may be commended to students and practitioners as a sober and trustworthy guide on the subjects of which it treats.

### HOME REMEDIES.

On a stiff card, less than two feet square, which can be hung up in the nursery, the schoolroom, the shop, or the kitchen, we have what may be called a *Medical Chart*, which lets us know exactly where we are in almost every conceivable medical or surgical latitude. The idea is excellent, and as excellently carried out. Are you bitten by a dog or a snake; have you swallowed a pin or a sovereign; has a midge invaded your eye, or an earwig your ear; have you got a burn or a scald or a black eye; in fine, have any of the thousand and one casualties of infantile, school, or adult life fallen upon you? Consult your chart, and you know exactly what to do *for* the moment *in* a moment, and then you can wait with patience "until the Doctor comes." The price is a shilling. The authoress, we understand, is Mrs. Coates, and the chart can be purchased at any of the Stores.

